# Case report: Nerve fiber regeneration in children with melanocortin 4 receptor gene mutation related obesity treated with semaglutide

**DOI:** 10.3389/fendo.2024.1385463

**Published:** 2024-06-21

**Authors:** Hoda Gad, Idris Mohammed, Hajar Dauleh, Maheen Pasha, Tara Al-Barazenji, Khalid Hussain, Rayaz A. Malik

**Affiliations:** ^1^ Research Department, Weill Cornell Medicine-Qatar, Doha, Qatar; ^2^ Endocrinology Department, Sidra Medicine, Doha, Qatar; ^3^ College of Health & Life Sciences, Hamad Bin Khalifa University, Doha, Qatar; ^4^ Institute of Cardiovascular Medicine, University of Manchester, Manchester, United Kingdom

**Keywords:** monogenic obesity, neurodegeneration, nerve regeneration, GLP-1 - glucagon-like peptide-1, semaglutide

## Abstract

Melanocortin 4 receptor (*MC4R*) mutations are the commonest cause of monogenic obesity through dysregulation of neuronal pathways in the hypothalamus and prefrontal cortex that regulate hunger and satiety. *MC4R* also regulates neuropathic pain pathways via JNK signaling after nerve injury. We show evidence of corneal small fiber degeneration in 2 siblings carrying a heterozygous missense variant c.508A>G, p.Ille170Val in the *MC4R* gene. Both children were treated with once weekly semaglutide for 6 months with no change in weight, and only a minor improvement in HbA1c and lipid profile. However, there was evidence of nerve regeneration with an increase in corneal nerve fiber density (CNFD) [child A (13.9%), child B (14.7%)], corneal nerve branch density (CNBD) [child A (110.2%), child B (58.7%)] and corneal nerve fiber length (CNFL) [child A (21.5%), child B (44.0%)].

## Introduction

Obesity is a multifactorial disease due to genetic predisposition and environmental factors. Whilst polygenic variants are frequent, they confer small effect sizes, whilst rare pathogenic variants in single genes with large effect sizes account for ~5% of pediatric obesity (1). Clinically, patients with monogenic obesity present with impaired satiety and hyperphagia in early childhood with severe early-onset obesity due to dysregulation of the central leptin-melanocortin neuronal pathways (2, 3). We have recently characterized two novel homozygous variants that yielded antagonistic proteins to leptin receptor activation resulting in intense hyperphagia and severe obesity in one child in Qatar (4). We have also identified pathogenic variants in around 14.8% of 243 individuals with early-onset obesity, in whom variants in the MC4R gene accounted for 19% (2), far higher than other studies (0.5–8.5%) (5). MC4R is expressed on neurons in the hypothalamus and prefrontal cortex, which regulate hunger and satiety (6, 7).

Lifestyle interventions in patients with *MC4R* mutations have shown minimal benefit (8–10) and even bariatric surgery has shown limited long-term benefit (11–13). Targeted agonism of the *MC4R* with setmelanotide showed ~10% body weight loss in patients with *POMC* deficiency and *LEPR* deficiency (14), and there are case reports showing weight loss after GLP-1 therapy in individuals with pathogenic variants of *MC4R* (15–17). A recent case report of a 13-year-old female with a heterozygous c.206T>G(p.I69R) variant in the *MC4R* gene showed that treatment with liraglutide achieved a 19.2% reduction in body weight at 32 weeks, but significant gastrointestinal side-effects led to cessation of treatment and a return to her former weight within 13 weeks (18).

GLP-1 receptor agonism leads to improvements in weight, blood pressure, lipids with a decrease in reactive oxygen species (ROS) and inflammation which could impact on neurons (19). Indeed, GLP-1R-mediated ERK-signaling in diabetic rodents has been shown to protect large motor fiber function and small fiber structure by a mechanism independent of glycemic control (20). We have previously shown corneal nerve regeneration after bariatric surgery (21) and after treatment with the once weekly GLP-1 agonist exenatide (22). More recently, in 14 patients with type 2 diabetes, treatment with semaglutide or dulaglutide improved nerve area and sural nerve amplitude (23). We have now undertaken corneal confocal microscopy to assess for evidence of nerve regeneration following treatment with the once weekly GLP-1 RA semaglutide, in two siblings with MC4R gene mutation.

## Methods

Two siblings, a 10-year-old boy (patient A) and an 8-year-old girl (patient B) with intense hyperphagia, impaired satiety, and severe, early onset obesity and a 10-year-old healthy boy were studied.

### Anthropometry

Weight (kg) was measured using the body composition analyzer (TANITA DC-430MAIII) and height (cm) using the stadiometer (SECA model), both were recorded to the nearest 0.1 g or cm, respectively (24). The cut-off points to classify weight status were established using the International Obesity Task Force (IOFT) (25) and World health Organization (WHO) growth chart (26). BMI is a poor predictor of adiposity due to the indirect relationship to fat content (27), and we have therefore additionally assessed body composition using the TANITA scale to derive body fat percent (BF%) and fat mass (kg).

### Cardiometabolic panel assessments

Glycated hemoglobin (HbA1c), total cholesterol (TC), LDL cholesterol (LDL-C), HDL cholesterol (HDL-C), and triglycerides (TG) were assessed.

### Neuropathy and neuropathic pain assessments

#### Vibration perception threshold

The stimulator was applied on the pulp of both big toes, and the stimulus strength increased slowly from zero until the vibration was first perceived by indicating “yes”. Vibration sensation was recorded as an average for both feet in volts (28). A VPT of *≥* 15V was considered to be impaired vibration perception (29).

#### Monofilament

A 10 g monofilament (Semmes-Weinstein monofilament) was applied with a sufficient force to cause the filament to bend at a total of 9 sites per foot, on both feet. Loss of protective sensation was recorded as “no feeling in *≥* 8 sites” (30).

#### Corneal confocal microscopy

Corneal confocal microscopy was undertaken in all three children using the Heidelberg Retina Tomograph III Rostock Cornea Module (Heidelberg Engineering, Heidelberg, Germany). Both eyes were anaesthetized with 2 drops of Bausch & Lomb Minims ^®^ (Oxybuprocaine hydrochloride 0.4% w/v). A drop of hypotears gel (Carbomer 0.2% eye gel) was placed on the tip of the objective lens and a sterile disposable TomoCap was placed over the lens, allowing optical coupling of the objective lens to the cornea. Six images were selected from the sub basal nerve plexus (SBNP) in the central cornea and corneal nerve fiber density (CNFD) (fibers/mm^2^) corneal nerve branch density (CNBD) (branches/mm^2^), and corneal nerve fiber length (CNFL) (mm/mm^2^) were quantified manually using CCMetrics. The investigator was blind to the study group when analyzing the CCM images.

### Douleur neuropathique en 4 questionnaire

Neuropathic pain was assessed using the Douleur Neuropathique en 4 (DN4) questionnaire which can distinguish neuropathic from non-neuropathic pain. The diagnosis of painful neuropathy was based on a DN4 questionnaire score of ≥4, which has a high sensitivity (80%) and specificity (92%) for diagnosing painful diabetic neuropathy in adults (31).

## Results

Clinical demographics of the cases are described in **Table 1**. Child A weighed 100.3kg and child B weighed 58.6kg. Both siblings carry a heterozygous missense variant c.508A>G, p.Ille170Val in the MC4R gene, a rare variant with Minor allele frequency (MAF) in gnomAD of 0.00009. The variant is predicted to have PM1/PM2/PP3/PP5/BS2 classes, which is classified as a pathogenic variant according to the ACMG variant classification. It is located in the transmembrane helix of the MC4R gene and is known to impair cyclic-AMP, leading to severe obesity (32, 33).

**Table 1:** Body composition, metabolic and CCM variables before and after semaglutide treatment.

Variable	Patient A		Patient B	
	Before treatment	6-m after treatment	Before treatment	6-m after treatment
Weight (kg)				
Value	100.3	100.9	58.6	57.4
Δ from baseline (%)	0.6		-2.1	
Body-mass index (kg/m^2^)				
Value	39.7	37.1	32.2	28.5
Δ from baseline (%)	-6.5		-11.5	
Body fat (%)				
Value	50.4	53.8	47.7	42.6
Δ from baseline (%)	6.8		-10.7	
Fat mass (kg)				
Value	50.6	54.3	28.0	24.5
Δ from baseline (%)	7.3		-12.5	
HbA1c (%)				
Value	5.8	5.5	5.6	5.4
Δ from baseline (%)	-5.2		-3.6	
Total cholesterol (mmol/L)				
Value	4.4	3.8	3.4	3.6
Δ from baseline (%)	-13.6		5.9	
LDL-C (mmol/L)				
Value	2.3	2.3	2	2.3
Δ from baseline (%)	0.0		15.0	
HDL-C (mmol/L)				
Value	2.1	1.5	1	1.3
Δ from baseline (%)	-28.6		30.0	
TG (mmol/L)				
Value	1	0.8	1.4	1.2
Δ from baseline (%)	-20.0		-14.3	
CNFD (fiber/mm^2^)				
Value	30.2	34.4	35.4	40.6
Δ from baseline (%)	13.9		14.7	
CNBD (branch/mm^2^)				
Value	31.2	65.6	35.4	56.2
Δ from baseline (%)	110.2		58.7	
CNFL (mm/mm^2^)				
Value	18.6	22.6	19.3	27.8
Δ from baseline (%)	21.5		44.0	
VPT (V)				
Value	3	1.8	1.5	5.5
Δ from baseline (%)	-40.0		266	
Monofilament				
Value	10	10	10	10
Δ from baseline (%)	No change		No change	

LDL-C, low-density lipoprotein cholesterol; HDL-C, high-density lipoprotein cholesterol; TG, triglycerides; CNFD, corneal nerve fiber density; CNBD, corneal nerve branch density; CNFL, corneal nerve fiber length; VPT, vibration perception threshold.

### GLP-1 treatment

Both children with obesity were treated with once weekly semaglutide 0.5mg for 1 month and then 1.0 mg once weekly for 5 months.

### Clinical/anthropometric and metabolic variables

Body weight increased in child A (0.6%) and decreased in child B (-2.0%). BMI decreased in child A (-6.5%) and child B (-11.5%) due to an increase in height (child A +3.8% and child B +5.2%). Whereas percentage body fat (6.8%) and fat mass (7.3%) increased in child A and percentage body fat (-10.7%) and fat mass (-12.5%) decreased in child B (**Table 1**). There were reduction in HbA1c in child A (-5.2%) and child B (-3.6%), total cholesterol decreased in child A (-13.6%) and increased in child B (5.9%), LDL did not change in child A (0%) and increased in child B (5.9%), HDL decreased in child A (-28.6%) and increased in child B (30.0%), whilst triglycerides decreased in both child A (-20.0%) and child B (-14.3%).

### Neuropathy measures

Vibration perception threshold was normal in child A (3.0V) and child B (1.5V) and decreased in child A (1.8V, -40%) but increased in child B (5.5V, +266%). Sensation to the monofilament at all 8 sites on the foot was normal at baseline and did not change at follow up. Both children scored zero for the DN4 questionnaire at baseline and after treatment.

**Figure 1:**
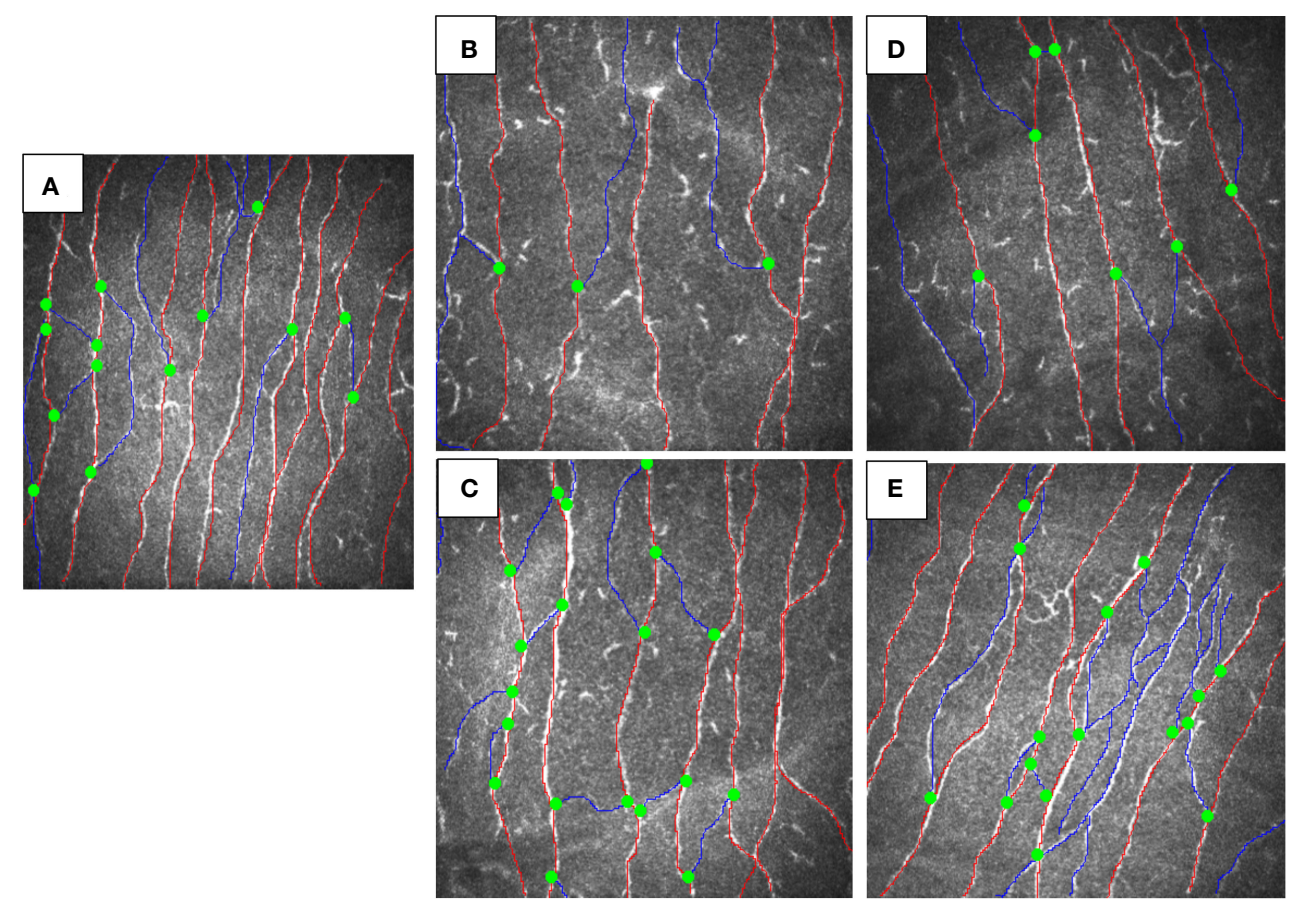
CCM images of siblings with MC4R mutation before and after treatment vs. healthy control. **(A)** healthy control, **(B)** child A before treatment, **(C)** child A after 6-months treatment with GLP-1, **(D)** child B before treatment, **(E)** child B after 6-months treatment with semaglutide.

**Figure 2:**
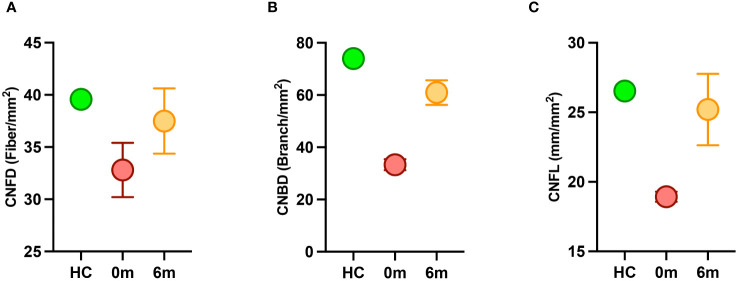
Corneal nerve parameters in children with MC4R mutation before (red) and after (orange) treatment with semaglutide compared to a healthy control (HC) (green). **(A)** CNFD, corneal nerve fiber density; **(B)** CNBD, corneal nerve branch density; (C) CNFL, corneal nerve fiber length.

Both siblings had a lower CNFD (child A-30.2 fiber/mm^2^, child B-35.4 fiber/mm^2^ vs. HC 39.6 fiber/mm^2^), CNBD (child A-31.2 branch/mm^2^, child B-35.4 branch/mm^2^ vs. HC-73.9 branch/mm^2^), and CNFL (child A-18.6 mm/mm^2^, child B-19.3 mm/mm^2^ vs. HC 26.53 mm/mm^2^) compared to the healthy control (**Figures 1A, B, D**). After 6 months of treatment with semaglutide, there was evidence of nerve regeneration (**Figures 1A–E**) with an increase in CNFD [child A (13.9%), child B (14.7%)] (**Figure 2A**), CNBD [child A (110.2%), child B (58.7%)] (**Figure 2B**) and CNFL [child A (21.5%), child B (44.0%)] (**Figure 2C**).

## Discussion

This study shows evidence of sub-clinical nerve degeneration with regeneration following treatment with the GLP-1 agonist semaglutide in two children with a *MC4R* gene mutation and severe obesity. First, we show evidence of corneal small nerve fiber degeneration, with preserved vibration perception and sensation to the 10g monofilament, and normal DN4 indicative of subclinical neurodegeneration, detected with corneal confocal microscopy. Clinical neuropathy has been reported in a 27-year old male with a *MC4R* gene mutation and morbid obesity, but this was attributed to the presence of T2DM (34).

We also show that 6 months of treatment with semaglutide was associated with small nerve fiber regeneration, but with a no major effect on weight, HbA1c and lipids, arguing for alternate mechanisms beyond an improvement in weight and glycemia as a basis for nerve regeneration in these two children with MC4R gene mutation. Obesity perse is a risk factor for small fiber neuropathy (35, 36) and we have previously shown nerve regeneration with an improvement in weight and metabolic parameters after bariatric surgery (37, 38). Furthermore, GLP-1 receptor agonists reduce weight and improve many of the risk factors for neuropathy including hyperglycemia, blood pressure and hyperlipidemia (39). Indeed, previous case reports with the daily GLP-1 agonist liraglutide have shown a reduction in weight and improvement in glycemia (15–17). However, in our recent cohort study we showed no major impact of liraglutide on weight or HbA1c in children with obesity (40).

There is a body of evidence that *MC4R* mediates neuropathic (41) and inflammatory pain (42) and the *MC4R* antagonist HS014 has been shown to increase paw withdrawal threshold and heat withdrawal latency in rat models of neuropathic pain (43). In the present study there was no evidence of neuropathic pain based on the DN4 score in children with MC4R mutation and it did not change with semaglutide. DN4 has not been validated in children with obesity, but it has been used to assess neuropathic pain in children with leprosy-related neuropathic pain (44) and sickle cell disease (45).

With regard to alternate mechanisms for nerve regeneration, sirtuin-1 (SIRT-1) inactivation has been implicated in obesity and neurodegeneration and SIRT-1 activation is associated with nerve regeneration following peripheral nerve injury (46, 47). Of note, GLP-1 therapies are SIRT-1 activators (48, 49) which may be associated with the nerve regeneration observed in our patients, independent of change in weight and glycemia. Additionally, GLP-1 receptors are expressed in the dorsal root ganglion and peripheral nerves (50–53) and in a T1DM animal model, GLP-1 treatment led to intraepidermal nerve fiber regeneration without a change in weight or glucose (20). Furthermore, previously, in adults with T2DM treated with once weekly exenatide and pioglitazone, we showed evidence of small nerve fiber regeneration, despite an increase in weight (22).

We acknowledge this is a study of only two children with the MC4R mutation with a limited duration of follow up after treatment with semaglutide. However, we believe that our study provides novel insights into the complications associated with MC4R gene mutation as evidenced by subclinical neurodegeneration. Furthermore, we show nerve regeneration after treatment with semaglutide, without an improvement in weight or glycemia, indicating an independent effect of GLP-1 therapy, which merits further study.

## Data availability statement

The original contributions presented in the study are included in the article/supplementary materials, further inquiries can be directed to the corresponding author/s.

## Ethics statement

The studies involving humans were approved by Sidra Medicine and WCM-Q IRB committees. The studies were conducted in accordance with the local legislation and institutional requirements. Written informed consent for participation in this study was provided by the participants’ legal guardians/next of kin. Written informed consent was obtained from the individual(s), and minor(s)’ legal guardian/next of kin, for the publication of any potentially identifiable images or data included in this article.

## Author contributions

HG: Writing – original draft, Project administration, Methodology, Investigation, Formal analysis, Data curation, Conceptualization. IM: Writing – review & editing, Data curation. HD: Writing – review & editing, Data curation. MP: Writing – review & editing, Data curation. TA: Writing – review & editing, Data curation. KH: Writing – review & editing, Supervision. RM: Writing – review & editing, Supervision.
